# Association between Consumption of Iodine-Rich Foods and Thyroid Cancer Prevalence: Findings from a Large Population-Based Study

**DOI:** 10.3390/nu16071041

**Published:** 2024-04-03

**Authors:** Yu-Jin Kwon, Hye-Sun Lee, Sang-Wook Kang, Ji-Won Lee

**Affiliations:** 1 Department of Family Medicine, Yongin Severance Hospital, Yonsei University College of Medicine, Yongin 16995, Republic of Korea; digda3@yuhs.ac; 2 Biostatistics Collaboration Unit, Department of Research Affairs, Yonsei University College of Medicine, Seoul 03722, Republic of Korea; hslee1@yuhs.ac; 3 Department of Surgery, Yonsei University College of Medicine, Seoul 03722, Republic of Korea; 4 Department of Family Medicine, Severance Hospital, Yonsei University College of Medicine, Seoul 03722, Republic of Korea; 5 Institute for Innovation in Digital Healthcare, Yonsei University, Seoul 03722, Republic of Korea

**Keywords:** thyroid cancer, iodine-rich foods, eggs, seaweeds, dairy products, population-based study

## Abstract

The influence of iodine-rich foods on thyroid cancer (TC) risk remains inadequately understood. Therefore, we aimed to comprehensively investigate the relationship between three iodine-rich food groups and TC prevalence using extensive data from a large Korean population. We assessed the dietary intake of 169,057 participants in the Korean Genome and Epidemiology Study (2004–2013) using a food frequency questionnaire. The top-three iodine-rich food groups (including egg, seaweed, and dairy) were selected based on Korean dietary reference intakes and categorized by weekly consumption frequency. We conducted multiple logistic regression models to examine the relationship between food consumption and TC prevalence. After adjusting for confounding factors, higher seaweed consumption (>5 times/week) was significantly associated with lower TC prevalence (odds ratio [OR], 95% confidence interval [CI] = 0.42, 0.32–0.56, *p*-value < 0.001). In contrast, compared with moderate dairy consumption (3–4 times/week), lower dairy product intake (<1 time/week) was associated with higher TC prevalence (OR, 95% CI = 1.32, 1.05–1.67, *p*-value = 0.017). Our findings suggest that sufficient seaweed consumption may offer protection against TC, and incorporating dairy products into the diet may lower TC incidence in the Korean population. The most significant limitations of our study are the absence of 24 h urine samples for iodine status assessment and the lack of clinical data on the diagnosis of thyroid cancer.

## 1. Introduction

Thyroid cancer (TC), a prevalent endocrine malignancy, has exhibited a substantial global rise over several decades, with considerable variability in the incidence rates between and within countries [1,2]. Mortality rates associated with TC have remained relatively stable or even decreased; however, South Korea is witnessing the highest incidence rate, reaching 45 cases per 100,000 individuals [2]. The high incidence of TC in Korea can be partially attributed to issues of over-diagnosis, but the substantial medical costs associated with it cannot be disregarded. Moreover, public interest in TC remains pronounced [3]. TC etiology is complex and influenced by a combination of genetic predisposition and environmental factors [4,5].

Numerous studies have sought to elucidate the link between diet and TC [6,7,8]. Specific evidence suggests that dietary patterns and the risks associated with certain nutrients, such as iodine, could influence the modulation of TC risk [9,10]. Iodine, an essential trace element, is important for the synthesis of thyroid hormones and the maintenance of thyroid function [11]. Iodine deficiency and excessive iodine intake can contribute to an increased prevalence of benign thyroid conditions and, plausibly, an elevated risk of TC [11,12]. However, the influence of iodine-rich foods on TC risk remains relatively poorly understood.

The influence of geographical, socioeconomic, ethnic, and cultural factors must be considered in studying TC risk factors [4,5]. Additionally, the complexities of the relationship between dietary consumption and TC occurrence should be considered, as this relationship is influenced by the presence of various bioactive compounds in foods, aside from the multifaceted effects of iodine [6,7,8].

Therefore, we aimed to comprehensively investigate the relationship between three iodine-rich food groups and TC prevalence using extensive data from a large Korean population. First, we identified the three primary food groups that contribute significantly to iodine intake among the Korean population. Subsequently, we explored the potential association between the consumption of specific iodine-rich foods, namely eggs, seaweeds, and dairy products, and TC prevalence using a large population-based study. Our research aimed to enhance the comprehension of dietary factors that could influence TC prevalence by examining the intricate relationship between the consumption of iodine-rich foods and the potential risk of TC.

## 2. Materials and Methods

### 2.1. Participants

We conducted an analysis using data from adult participants aged 40 years and older who were part of the Korean Genome and Epidemiology Study (KoGES) Health Examinee Study conducted between 2004 and 2013. The KoGES is a large-scale population-based study. Detailed information about the KoGES can be found in a previous study [13] and is also available on the following website: https://www.kdca.go.kr/contents.es?mid=a40504010000 (1 August 2023).

**Figure 1:**
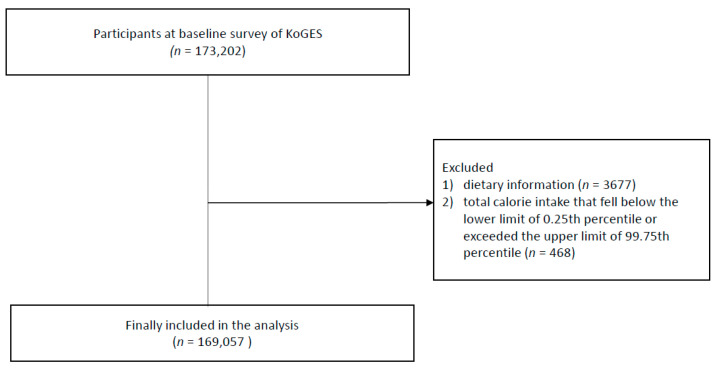
Flow chart.

Of the 173,202 participants included in the KoGES between 2001 and 2013, we included 169,057 participants after excluding 4145 individuals without dietary information (*n* = 3677) and those with total calorie intake below the lower limit of the 0.25th percentile or exceeding the upper limit of the 99.75th percentile (*n* = 468) (Figure 1). Informed consent was obtained from all participants, and the institutional review board (IRB) of Yongin Severance Hospital (IRB number: 3-2020-0043) approved this study to ensure ethical compliance.

### 2.2. Dietary Assessment

Dietary intake was evaluated using a semi-quantitative food frequency questionnaire (FFQ), which was administered by a trained interviewer. This FFQ was specifically designed for the community-based cohort of the KoGES [14]. It captured the frequency of food consumption for each participant over the previous year. Based on the Korean dietary reference intakes, we selected the top-three food groups (including egg, seaweed, and dairy products) as the primary sources of iodine in the Korean population [15]. Each food item was categorized based on the weekly consumption frequency as follows: less than 1 time a week, 1–2 times/week, 3–4 times/week, and 5 or more times/week. Nutrient (macro-/micro-nutrient) consumption was assessed using the FFQ.

### 2.3. Covariates

Trained medical personnel conducted all health examination procedures. The body mass index (BMI) was calculated by dividing an individual’s weight in kilograms by the square of their height in meters. Blood pressure measurements were obtained twice while the participants were seated. Blood tests were conducted following an 8 h fasting period. Serum glucose, glycated hemoglobin, total cholesterol, high-density lipoprotein cholesterol (HDL-C), triglyceride, and C-reactive protein (CRP) levels were determined enzymatically using a Chemistry Analyzer (ADVIA 1650, Siemens, Tarrytown, NY, USA). Smoking status, alcohol intake status, physical activity (regular exercisers, defined as individuals who engaged in regular exercise resulting in sweating), and household income were self-reported through the questionnaire. Conditions such as hypertension (HTN), diabetes mellitus (DM), and dyslipidemia were recorded as binary variables.

### 2.4. Study Outcomes

The presence of TC was investigated through a questionnaire to determine if individuals had a history of experiencing this condition. When participants self-reported having a specific disease, interviewers double-checked whether the respondent had received their diagnosis from a doctor to improve the reliability of our survey.

### 2.5. Statistical Analysis

Data are presented as means (standard deviation) and numbers (percentages). Data were compared using one-way analysis of variance for continuous variables or the chi-square test for categorical variables. Restricted cubic spline curve analyses were developed and applied in an unadjusted model. Univariable and multivariable logistic regression models were constructed to evaluate the independent relationship between egg, seaweed, and dairy product consumption and TC prevalence. In the multivariable logistic regression models, we adjusted for variables with a *p*-value < 0.1 in the univariate analysis, as well as previously reported variables, including age, sex, BMI, alcohol intake, smoking, regular exercise, and total calorie intake. All statistical analyses were conducted using SAS 9.2 (SAS Institute, Cary, NC, USA). Statistical significance was set at two-sided *p*-values < 0.05.

## 3. Results

### 3.1. Clinical Characteristics of the Study Population

Of the 169,057 participants, 930 (0.55%) had TC. Table 1 presents the clinical characteristics of the study population based on the presence of TC. Most patients with TC were women (91.9%). Significant differences were observed in waist circumference (WC), systolic blood pressure (SBP), diastolic blood pressure (DBP), fasting glucose, total cholesterol, and triglyceride levels, as well as smoking status, alcohol consumption, physical activity, the prevalence of underlying diseases such as HTN, dyslipidemia, and DM, and household income.

Table 2 presents the clinical characteristics of the study population based on the frequency of egg, seaweed, and dairy product consumption. Compared with those who consumed eggs less than 1 time/week, those who consumed eggs more than 5 times/week were more likely to be male and younger. They also had lower BMI, WC, SBP, DBP, fasting glucose, triglyceride, and CRP levels. Moreover, compared with participants who consumed eggs less than 1 time/week, those who consumed eggs more than 5 times/week had lower proportions of smoking and HTN, dyslipidemia, and DM. Furthermore, participants who consumed eggs more than 5 times/week engaged in more exercise and had higher alcohol intake and household income than those who consumed eggs less than 1 time/week. Regarding dietary intake, participants who consumed eggs more than 5 times/week consumed more total calories (kcal/day), carbohydrates (g/day), fats (g/day), proteins (g/day), and micro-nutrients than those who consumed eggs less than 1 time/week. However, compared with participants who consumed eggs less than 1 time/week, those who consumed eggs more than 5 times/week had lower proportions of carbohydrates in their diet and higher proportions of fats and proteins.

Compared with participants who consumed seaweeds less than 1 time/week, those who consumed seaweeds more than 5 times/week were more likely to be female and older. They also had lower WC, SBP, fasting glucose, triglyceride, and CRP levels, but higher total cholesterol and HDL-C. Participants who consumed seaweeds more than 5 times/week had lower proportions of smoking, alcohol intake, and DM but exercised more, had higher proportions of dyslipidemia, and had lower household income than those who consumed seaweeds less than 1 time/week. Regarding dietary intake, participants who consumed seaweeds more than 5 times/week consumed higher amounts of total calories and macro-/micro-nutrients than those who consumed seaweeds less than 1 time/week. The proportion of carbohydrates in their diet was lower, whereas the proportions of fats and proteins were higher than those of the participants who consumed seaweeds less than 1 time per week.

Participants who consumed dairy products more than 5 times/week were more likely to be female and younger, with lower BMI, WC, SBP, DBP, fasting glucose, and triglyceride levels, whereas they had higher total cholesterol and HDL-C. Participants who consumed dairy products more than 5 times/week had higher proportions of smoking, were regular exercisers, and had dyslipidemia, but had a lower proportion of alcohol intake and a lower incidence of HTN and DM compared with those who consumed dairy products less than 1 time/week. Regarding dietary intake, participants who consumed dairy products more than 5 times/week consumed higher amounts of total calories and macro-/micro-nutrients than those who consumed dairy products less than 1 time/week. The proportion of carbohydrates in their diet was lower, whereas the proportion of fats and proteins was higher than that of those who consumed dairy products less than 1 time/week.

### 3.2. Association between Iodine-Rich Food Consumption and TC Prevalence

**Figure 2:**
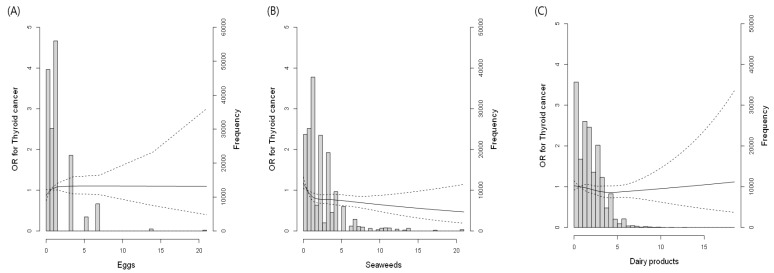
Density plots and restricted cubic spline curves on the relationship between the frequency of egg (**A**), seaweed (**B**), and dairy product (**C**) consumption and thyroid cancer prevalence.

Figure 2A–C present density plots and restricted cubic spline curves illustrating the relationship between the frequency of egg, seaweed, and dairy product consumption and TC prevalence. The cubic spline curves revealed no discernible link between egg consumption and TC prevalence. We observed an inverse association between seaweed intake and TC and a reverse J-shaped relationship between dairy product consumption and TC.

Table 3 displays the outcomes of multiple logistic regression analyses investigating the connection between each food intake and TC. In the unadjusted model, the frequency of egg consumption did not significantly correlate with TC prevalence (≥5 times/week vs. <1 time/week, odds ratio [OR], 95% confidence interval [CI] = 1.03, 0.78–1.37, *p*-value = 0.84). This result remained after adjusting for confounders.

**Figure 3:**
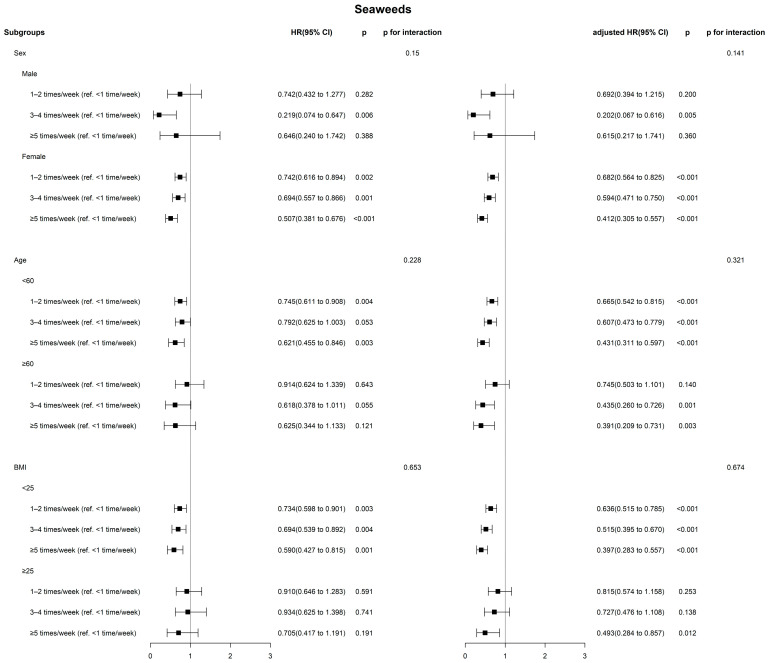
Association between seaweed consumption and thyroid cancer based on age, sex, and obesity groups.

In the unadjusted model, the frequency of seaweed consumption was associated with TC prevalence (<1 time/week vs. ≥5 times/week, hazard ratio [HR], 95% CI = 0.62, 0.47–0.82, *p*-value < 0.001). After controlling for confounders, the group that consumed seaweeds more than 5 times/week exhibited a lower risk of TC than the group consuming seaweeds less than 1 time/week (HR, 95% CI = 0.42, 0.32–0.56, *p*-value < 0.001). Compared with the group that consumed dairy products 3–4 times/week, the group that consumed dairy products less than 1 time/week demonstrated a higher TC prevalence in the unadjusted model (OR, 95% CI = 1.31, 1.05–1.63, *p*-value = 0.017). This significant association remained after adjusting for confounders. Figure 3 and Supplementary Figures S1 and S2 present the results of a subgroup analysis based on sex, age group, and obesity status.

No significant associations were observed between egg consumption and TC prevalence based on sex, age group, and obesity status in any of the subgroups (Supplementary Figure S1). Figure 3 shows the subgroup analysis based on sex, age group, and obesity status in relation to seaweed consumption. Compared to the group consuming seaweeds less than 1 time/week, consuming seaweed more than 5 times/week was associated with lower TC prevalence in women, participants aged under 60 years, and those with a BMI lower than 25. In the unadjusted model, there was a very slight trend toward significance between seaweed consumption and TC among men, participants aged 60 years and older (*p* = 0.121), and those with a BMI of 25 and above (*p* = 0.191). After adjusting for confounders, higher seaweed consumption was associated with lower TC prevalence in individuals aged 60 years and older (*p* = 0.003), as well as in the group of participants with a BMI of 25 and above (*p* = 0.012). Supplementary Figure S2 presents subgroup analyses based on sex, age group, and obesity status concerning dairy consumption. Among women, the adjusted ORs with 95% CI indicated that the group consuming dairy products less than 1 time/week had an OR of 1.319 (1.04–1.67) for TC compared with those who consumed dairy products 3–4 times per week. Furthermore, in the age group younger than 60 years and the group with a BMI lower than 25, consuming dairy products less than 1 time/week was associated with a higher TC prevalence than in those who consumed dairy products 3–4 times/week. However, no significant associations were observed between dairy product consumption and TC prevalence in the group aged 60 years and younger and the group with a BMI lower than 25.

## 4. Discussion

We comprehensively investigated the relationship between three iodine-rich food groups and TC prevalence using extensive data from a large Korean population. Our study revealed no significant association between egg consumption and TC prevalence. Conversely, we observed an inverse relationship between seaweed intake and TC and a reverse J-shaped pattern in relation to dairy product consumption.

East Asia, including Korea, Japan, and China, is characterized by high iodine intake, primarily from seafood consumption, especially seaweed [16,17]. Recent cohort studies have suggested a potential link between seaweed consumption and a reduced risk of cardiovascular diseases, possibly through blood pressure- and lipid-lowering effects [18,19]. However, the evidence regarding the influence of seaweed on cancer is limited and inconsistent. For instance, the Japan Public Health Center-Based Prospective Study discovered that a higher frequency of seaweed intake was associated with increased papillary TC in postmenopausal women but not in premenopausal women [20]. In contrast, the Japan Collaborative Cohort Study indicated that seaweed intake was not linked to TC incidence among premenopausal and postmenopausal women [21]. We observed that higher consumption of seaweed is associated with lower TC prevalence. Many studies have attempted to establish a relationship between dietary iodine consumption and TC prevalence to better understand the etiology underlying the increased risk of developing TC. Adequate iodine levels might offer protection against TC by promoting cell cycle arrest and apoptosis [12]. Moreover, chronic iodine deficiency is a risk factor for goiter and follicular TC. In contrast, excess iodine can inhibit thyroid hormone synthesis, increasing thyroid-stimulating hormone production and potentially causing goiter and papillary TC [22]. However, epidemiological results remain inconclusive. Some studies have suggested that higher iodine intakes are associated with an increased risk of TC [23], whereas others have demonstrated no such association [24,25] or even a potential protective effect against TC [26].

The exact reason for this relationship remains unknown; however, seaweed is not only rich in iodine but also contains various vitamins, minerals, dietary fibers, and flavonoids, considered preventive agents against lifestyle-related diseases [27]. Therefore, the effect of seaweed on TC is influenced by the interaction of various nutrients. Further research, including observational and interventional studies, is necessary to gain a comprehensive understanding of the influence of seaweed on TC.

Dairy products, also rich in iodine and essential nutrients, may protect against TC through various biological mechanisms [28]. These nutrients include calcium, vitamin D, and essential fatty acids. Specific dairy fatty acids exhibit anticancer properties by influencing cell growth and apoptosis genes [28]. Vitamin D can protect against tumors by binding to vitamin D receptors on TC cells, inhibiting their growth and impeding TC development [29]. Currently, the influence of dairy food intake on TC occurrence remains controversial [8,30,31]. A population-based case–control study conducted in two regions of Sweden and Norway reported that high cheese and butter intake is associated with higher TC risk [31]. However, a Japanese cohort study and an Italian case–control study have suggested that individuals with higher dairy intake had a lower TC risk [30,32]. A recent National Cancer Center survey in Korea discovered that consuming milk and dairy products more than 5 times/week is associated with a reduced risk of TC [8]. Our study identified a reverse J-shaped relationship with dairy consumption, aligning with previous research and showing an increased TC risk when consuming too few dairy products. Specifically, after accounting for confounding factors, the risk was relatively higher in the group that consumed dairy products less than 1 time a week than in the group that consumed dairy products 3–4 times/week. The disparities between the findings of previous studies and our study can be attributed to variations in the methods used to assess food consumption. Our study comprehensively evaluated 106 food items, including 337 foods that majorly contribute to 17 nutrients, including seasonal items, and was conducted by trained interviewers. In contrast, a prior National Cancer Center study utilized a dietary habit questionnaire consisting of only 12 items, potentially lacking a comprehensive assessment of all food items influencing TC development [8]. Confirmation of our findings will necessitate future studies, and investigating and considering specific dairy products, such as milk and butter, in more detail is essential.

In our study, eggs, a natural source of iodine in the human diet, were not associated with TC risk. Eggs are a highly nutritious food, and more than 60% of their calories come from fat—especially saturated fat, which concerns cardiovascular health [33]. The link between eggs and cancer remains poorly understood, with limited epidemiological studies focusing on their potential role in prostate and colon cancers [34,35]. Additionally, the iodine content in eggs varies depending on the diet of the chickens [33], underscoring the need for future research to measure iodine levels in eggs precisely and explore various cooking methods to better understand the relationship between eggs and TC.

Our study has some limitations. First, we could not establish causal relationships between specific iodine-rich foods and TC based on observational data. Second, our study population predominantly comprised women, despite TC exhibiting a robust female predominance. Third, relying solely on surveys rather than histological examination for the accurate diagnosis of thyroid cancer is indeed a significant limitation. Our analysis also lacks data on the histological subtypes of TC, although papillary TC is the most prevalent subtype, accounting for over 80% of cases in most countries [36]. Additional research is required to elucidate the molecular mechanisms through which iodine-rich foods may contribute to TC, particularly their specific effects on papillary and follicular TC. Additionally, we did not account for factors such as iodine-rich medications, multivitamins, and radiographic contrast agents [37]. Finally, despite the World Health Organization’s recommendation to use urine collections for accurately reflecting dietary iodine intake, we were unable to obtain the data regarding iodine intake through this method. The only available dataset in Korea that allows the use of disease codes is from the National Health Insurance Corporation (Daejeon, South Korea) which unfortunately does not include dietary or urinary iodine data. Therefore, establishing a cohort of thyroid cancer patients within a hospital setting to better elucidate these factors will be necessary in the future.

Nonetheless, our study has several strengths. One of its strengths is the use of a substantial sample size from a representative Korean database. Furthermore, it transcended the mere examination of the influence of iodine intake on TC risk. Instead, it delved into the relationship between the most common iodine-rich foods and TC, simultaneously considering the quality of these iodine-containing foods.

## 5. Conclusions

Our findings suggest that adequate seaweed consumption may offer protection against TC in the Korean population, and appropriate dairy product intake could be advantageous in reducing its incidence, potentially enhancing dietary habits for TC prevention in this population. In future research, collecting prospective data from large cohorts and conducting clinical studies that assess the influence of different iodine-rich foods on TC risk by measuring urinary iodine concentrations is imperative. 

## Figures and Tables

**Table 1:** Clinical characteristics of the study population.

	Non-Thyroid Cancer	Thyroid Cancer	*p*
n	168,127	930	
Men, n (%)	57,865 (34.4)	75 (8.06)	<0.001
Age (years)	53.1 ± 8.4	53.0 ± 7.5	0.811
Height (cm)	160.5 ± 8.0	158.5 ± 6.2	<0.001
Weight (kg)	61.8 ± 9.9	59.8 ± 8.5	<0.001
BMI (kg/m^2^)	23.9 ± 2.9	23.8 ± 2.9	0.134
Waist circumference (cm)	81.1 ± 8.7	79.3 ± 8.0	<0.001
Systolic BP (mmHg)	122.7 ± 15.5	120.7 ± 14.3	<0.001
Diastolic BP (mmHg)	76.2 ± 10.0	74.6 ± 9.4	<0.001
Glucose (mg/dL)	95.2 ± 21.7	93.6 ± 14.8	0.021
Total cholesterol (mg/dL)	197.5 ± 35.6	190.0 ± 35.2	<0.001
HDL-C (mg/dL)	54.0 ± 12.9	53.6 ± 13.1	0.387
Triglycerides (mg/dL)	127.0 ± 89.9	113.8 ± 66.0	<0.001
Smoking status (yes)	21,881 (13.0)	15 (1.6)	<0.001
Alcohol intake (yes)	82,626 (49.1)	283 (30.4)	<0.001
Regular exercise (yes)	87,955 (52.3)	549 (59.0)	
Hypertension (yes)	28,493 (17.0)	119 (12.8)	0.001
Diabetes mellitus (yes)	16,015 (9.5)	71 (7.6)	0.050
Dyslipidemia (yes)	94,744 (56.4)	448 (48.2)	<0.001
House income (KRW)			0.016
<1.5 million	30,344 (21.5)	151 (17.2)	
1.5–3.0 million	47,539 (33.68)	282 (32.9)	
3.0–6.0 million	51,509 (36.49)	342 (39.9)	
>6.0 million	11,771 (8.34)	82 (9.6)	
hs-CRP	0.15 ± 0.40	0.14 ± 0.3	0.434

Abbreviations: BMI, body mass index; BP, blood pressure; HDL-C, high-density lipoprotein cholesterol; hs-CRP, high-sensitivity C-reactive protein.

**Table 2:** Clinical characteristics of study participants based on the frequency of consumption of iodine-rich foods.

	Egg Consumption			Seaweed Consumption			Dairy Product Consumption		
	<1 Time/Week	≥5 Times/Week	*p*	<1 Time/Week	≥5 Times/Week	*p*	<1 Time/Week	≥5 Times/Week	*p*
n	47,572	13,003		23,720	17,113		35,672	4870	
Men, n (%)	15,114 (31.8)	4340 (33.4)	*	9604 (40.5)	4124 (24.1)	*	12,145 (34.1)	1503 (30.9)	*
Age (years)	55.2 ± 8.1	52.3 ± 8.6	*	53.3 ± 8.3	53.5 ± 8.3	*	54.2 ± 8.5	52.0 ± 8.0	*
BMI (kg/m^2^)	24.1 ± 2.9	23.7 ± 3.0	*	24.0 ± 2.9	23.9 ± 2.9	ns	24.0 ± 3.0	23.8 ± 2.8	*
WC (cm)	81.7 ± 8.5	80.2 ± 8.9	*	81.6 ± 8.7	80.4 ± 8.6	*	81.5 ± 8.7	80.1 ± 8.7	*
SBP (mmHg)	124.1 ± 15.7	121.1 ± 15.1	*	122.8 ± 15.7	122.4 ± 15.7	*	123.7 ± 15.8	120.7 ± 15.2	*
DBP (mmHg)	77.0 ± 10.0	75.5 ± 9.9	*	76.4 ± 10.2	76.3 ± 10.1	ns	76.7 ± 10.1	75.6 ± 9.9	*
Glucose (mg/dL)	96.2 ± 23.0	94.2 ± 19.4	*	95.6 ± 22.3	94.0 ± 21.2	*	96.6 ± 23.5	93.3 ± 20.4	*
TC (mg/dL)	197.8 ± 36.5	197.9 ± 35.3	ns	196.2 ± 35.5	199.4 ± 35.8	*	195.0 ± 36.2	202.4 ± 35.0	*
HDL-C (mg/dL)	53.1 ± 12.7	55.5 ± 13.6	*	53.3 ± 12.9	55.0 ± 13.1	*	52.9 ± 12.7	56.3 ± 13.5	*
TG (mg/dL)	130.3 ± 90.6	122.0 ± 88.5	*	129.3 ± 90.8	123.0 ± 85.6	*	133.2 ± 97.4	117.7 ± 82.0	*
hs-CRP	0.15 ± 0.40	0.14 ± 0.41	*	0.15 ± 0.42	0.14 ± 0.38	*	0.16 ± 0.47	0.14 ± 0.40	*
Smoking status	6571 (13.8)	1547 (11.9)	*	4037 (17.0)	1771 (10.4)	*	4530 (12.3)	616 (12.7)	*
Alcohol intake	21,130 (44.4)	6429 (49.4)	*	12,390 (52.2)	7292 (42.6)	*	16,473 (46.2)	7292 (42.6)	*
Regular exercise	23,977 (50.4)	7332 (56.4)	*	11,338 (47.8)	9999 (58.4)	*	17,586 (49.4)	2854 (58.9)	*
Hypertension	9299 (19.6)	1890 (14.5)	*	4192 (17.7)	2990 (17.5)	ns	6815 (19.1)	697 (14.3)	*
DM	5455 (11.5)	1007 (7.7)	*	2348 (9.9)	1523 (8.9)	*	4386 (12.3)	298 (6.1)	*
Dyslipidemia	27,506 (57.8)	7166 (55.1)	*	13,202 (55.7)	9846 (57.5)	*	19,810 (55.5)	2886 (59.3)	*
House income (KRW)			*			*			*
<1.5 million	11,275 (29.6)	1916 (17.0)		5052 (26.1)	2613 (18.9)		7923 (26.8)	634 (16.1)	
1.5–3.0 million	12,992 (34.2)	3669 (32.5)		6476 (33.4)	4725 (34.2)		10,108 (34.2)	1305 (33.2)	
3.0–6.0 million	11,319 (29.8)	4553 (40.4)		6342 (32.7)	5221 (37.8)		9483 (32.1)	1569 (39.9)	
>6.0 million	2449 (6.4)	1145 (10.2)		1509 (7.8)	1252 (9.1)		2042 (6.9)	424 (10.8)	
Energy (kcal/day)	1803.9 ± 622.8	2461.0 ± 818.7	*	1645.8 ± 570.0	2527.8 ± 838.8	*	1708.7 ± 574.5	2859.1 ± 921.1	*
CHO (g/day)	316.5 ± 100.2	386.9 ± 119.2	*	292.5 ± 93.2	401.0 ± 122.4	*	300.9 ± 93.8	441.3 ± 132.8	*
Fat (g/day)	32.7 ± 22.0	60.5 ± 32.1	*	28.3 ± 20.2	61.2 ± 33.0	*	29.7 ± 20.2	75.3 ± 36.6	*
Protein (g/day)	59.4 ± 27.8	92.6 ± 38.1	*	51.0 ± 23.3	97.1 ± 41.3	*	56.9 ±25.4	107.0 ± 45.1	*
CHO (%)	71.0 ± 7.7	63.6 ± 7.4	*	71.9 ± 7.8	64.3 ± 7.8	*	70.6 ± 7.9	62.5 ± 7.3	*
Fat (%)	15.5 ± 0.6	21.4 ± 5.6	*	14.7 ± 6.3	21.1 ± 5.7	*	15.5 ± 6.1	23.1 ± 5.4	*
Protein (%)	12.9 ± 2.4	14.9 ± 2.2	*	12.2 ± 2.2	15.2 ± 2.5	*	13.2 ± 2.3	14.7 ± 2.4	*
Na (mg/day)	2529.2 ± 1882.7	4054.2 ± 2487.1	*	1809.9 ± 1399.4	5158.8 ± 2818.8	*	2422.8 ± 20.2	4884.2 ± 3128.5	*
K (mg/day)	3526.0 ± 1885.9	5101.4 ± 2337.6	*	2721.8 ± 1474.9	5878.6 ± 2520.8	*	3237.7 ± 1717.4	6065.5 ± 2768.6	*
Ca (mg)	502.2 ± 303.3	789.1 ± 391.7	*	381.3 ± 237.7	909.6 ± 439.9	*	395.3 ± 218.2	1229.2 ± 507.1	*
P (mg)	1077.5 ± 479.7	1645.2 ± 637.6	*	908.3 ± 397.5	1739.5 ± 686.9	*	980.4 ± 417.4	2037.9 ± 750.3	*
Fe (mg)	17.2 ± 9.2	25.0 ± 12.1	*	14.0 ± 7.5	28.3 ± 13.2	*	16.5 ± 8.5	28.5 ± 14.7	*
Vitamin A (R.E.)	314.1 ± 247.3	551.7 ± 333.9	*	230.4 ± 184.8	631.4 ± 382.0	*	284.4 ± 216.8	706.7 ± 420.3	*
Niacin (mg)	12.9 ± 6.2	18.5 ± 7.9	*	10.6 ± 4.9	20.8 ± 8.8	*	12.2 ± 5.7	21.4 ± 9.6	*
Vitamin C (mg)	224.3 ± 188.9	311.0 ± 211.0	*	159.3 ± 156.3	366.4 ± 235.9	*	201.2 ± 173.2	369.5 ± 257.4	*
Zinc (μg)	11.4 ± 4.7	15.5 ± 5.9	*	9.7 ± 3.8	17.1 ± 6.6	*	12.2 ± 5.7	18.4 ± 7.1	*
Vitamin B6 (mg)	2.3 ± 1.7	3.3 ± 2.1	*	1.7 ± 1.2	3.9 ± 2.7	*	2.1 ± 1.5	3.8 ± 2.7	*
Folate (μg)	580.3 ± 304.4	872.3 ± 374.4	*	463.7 ± 247.2	956.4 ± 404.4	*	561.3 ± 286.0	953.6 ± 448.8	*
Retinol (μg)	55.5 ± 67.0	148.8 ± 91.6	*	53.1 ± 60.7	118.8 ± 108.2	*	38.1 ± 47.1	234.5 ± 140.8	*
Carotene (μg)	3103.2 ± 2599.5	4834.4 ± 3455.2	*	2127.7 ± 1874.4	6151.4 ± 3952.7	*	2955.4 ± 2367.5	5666.4 ± 4218.2	*
Fiber (g)	29.8 ± 15.6	42.7 ±19.4	*	23.0 ± 12.1	49.9 ± 21.0	*	28.6 ± 14.6	47.3 ± 23.4	*
Vitamin E (mg)	12.9 ± 8.2	21.9 ± 10.9	*	9.8 ± 6.2	24.6 ± 12.5	*	12.2 ± 7.5	24.6 ± 13.5	*
Cholesterol (mg)	99.2 ± 86.6	403.0 ± 167.7	*	113.5 ± 104.5	258.4 ± 179.0	*	127.9 ± 108.8	309.2 ± 195.3	*

Abbreviations: BMI, body mass index; WC, waist circumference; BP, blood pressure; TC, total cholesterol; TG, triglyceride; HDL-C, high-density lipoprotein cholesterol; hs-CRP, high-sensitivity C-reactive protein; DM, diabetes mellitus; CHO, carbohydrate; Na, sodium; K, potassium; Ca, calcium; P, phosphorus; Fe, iron; * denotes *p*-value < 0.05; ‘ns’ stands for ‘not significant’.

**Table 3:** Odds ratio and 95% confidence intervals for thyroid cancer based on egg, seaweed, and dairy product consumption.

	Unadjusted		Model 1		Model 2		Model 3	
	OR (95% CI)	*p*	OR (95% CI)	*p*	OR (95% CI)	*p*	OR (95% CI)	*p*
Egg consumption								
<1 time/week	0.87 (0.68–1.11)	0.268	0.86 (0.67–1.10)	0.223	0.88 (0.69–1.13)	0.307	0.89 (0.69–1.15)	0.361
1–2 times/week	0.99 (0.77–1.27)	0.938	1.02 (0.79–1.31)	0.901	1.03 (0.80–1.33)	0.808	1.04 (0.81–1.34)	0.771
3–4 times/week	1.03 (0.78–1.37)	0.84	1.05 (0.79–1.39)	0.759	1.05 (0.79–1.40)	0.713	1.06 (0.80–1.41)	0.696
≥5 times/week	ref		ref		ref		ref	
Seaweeds								
<1 time/week	ref		ref		ref		ref	
1–2 times/week	0.78 (0.65–0.93)	0.006	0.74 (0.62–0.88)	<0.001	0.72 (0.61–0.86)	<0.001	0.68 (0.57–0.82)	<0.001
3–4 times/week	0.75 (0.61–0.93)	0.01	0.66 (0.53–0.82)	<0.001	0.63 (0.51–0.79)	<0.001	0.57 (0.45–0.71)	<0.001
≥5 times/week	0.62 (0.47–0.82)	<0.001	0.51 (0.39–0.67)	<0.001	0.49 (0.37–0.64)	<0.001	0.42 (0.32–0.56)	<0.001
Dairy products								
<1 time/week	1.31 (1.05–1.63)	0.017	1.24 (0.99–1.55)	0.056	1.24 (0.99–1.56)	0.049	1.32 (1.05–1.67)	0.017
1–2 times/week	1.21 (1.00–1.47)	0.052	1.15 (0.95–1.40)	0.161	1.15 (0.94–1.39)	0.169	1.18 (0.97–1.43)	0.106
3–4 times/week	ref		ref		ref		ref	
≥5 times/week	1.25 (0.83–1.89)	0.282	1.16 (0.77–1.75)	0.486	1.14 (0.75–1.72)	0.539	1.12 (0.74–1.69)	0.588

Model 1: adjusted for age and sex. Model 2: adjusted for age, sex, and BMI. Model 3: adjusted for age, sex, BMI, smoking status, alcohol intake, exercise, and total calories. Abbreviations: CI, confidence interval; OR, odds ratio; BMI, body mass index.

## Data Availability

Data in this study were from the Korean Genome and Epidemiology Study (4851-302) and are available on the following website: https://www.kdca.go.kr/contents.es?mid=a40504010000 (1 August 2023).

## Supplementary Materials

The following supporting information can be downloaded at: https://www.mdpi.com/article/10.3390/nu16071041/s1, Supplementary Table S1. Common sources of iodine in the Korean diet, along with their iodine content (per 100 g); Supplementary Figure S1. Association between egg consumption and prevalence of thyroid cancer by sex, age, and obesity status; Supplementary Figure S2. Association between dairy product consumption and prevalence of thyroid cancer by sex, age, and obesity status.
