# Supplementary material for: Association between Consumption of Iodine-Rich Foods and Thyroid Cancer Prevalence: Findings from a Large Population-Based Study
Source: Nutrients. 2024 Apr 3;16(7):1041. doi: 10.3390/nu16071041 (PMC11013877; doi:10.3390/nu16071041)

Supplementary Materials

Association between Consumption of Iodine-Rich Foods and Thyroid Cancer Prevalence: Findings from a Large Population-Based Study

Yu-Jin Kwon [1], Hye-Sun Lee [2], Sang-Wook Kang [3,*] and Ji-Won Lee [4,5,*]

[1] Department of Family Medicine, Yongin Severance Hospital, Yonsei University College of Medicine, Yongin 16995, Republic of Korea
[2] Biostatistics Collaboration Unit, Department of Research Affairs, Yonsei University College of Medicine, Seoul 03722, Republic of Korea
[3] Department of Surgery, Yonsei University College of Medicine, Seoul 03722, Republic of Korea
[4] Department of Family Medicine, Severance Hospital, Yonsei University College of Medicine, Seoul 03722, Republic of Korea
[5] Institute for Innovation in Digital Healthcare, Yonsei University, Seoul 03722, Republic of Korea
* Correspondence: oralvanco@yuhs.ac (S.-W.K.); indi5645@yuhs.ac (J.-W.L.)

Supplementary Table S1. Common sources of iodine in the Korean diet, along with their iodine content (per 100 g).

	**Dietary Source**	**㎍/100g**
**1**	Roasted Seaweed	29,098
**2**	Seaweed	1,700
**3**	Egg	65
**4**	Milk	6
**5**	Anchovy	89
**6**	Kimchi	5
**7**	Snacks	36
**8**	Quail egg	240
**9**	Ice cream	22
**10**	Scallion	9

Supplementary Figure S1. Association between egg consumption and prevalence of thyroid cancer by sex, age, and obesity status

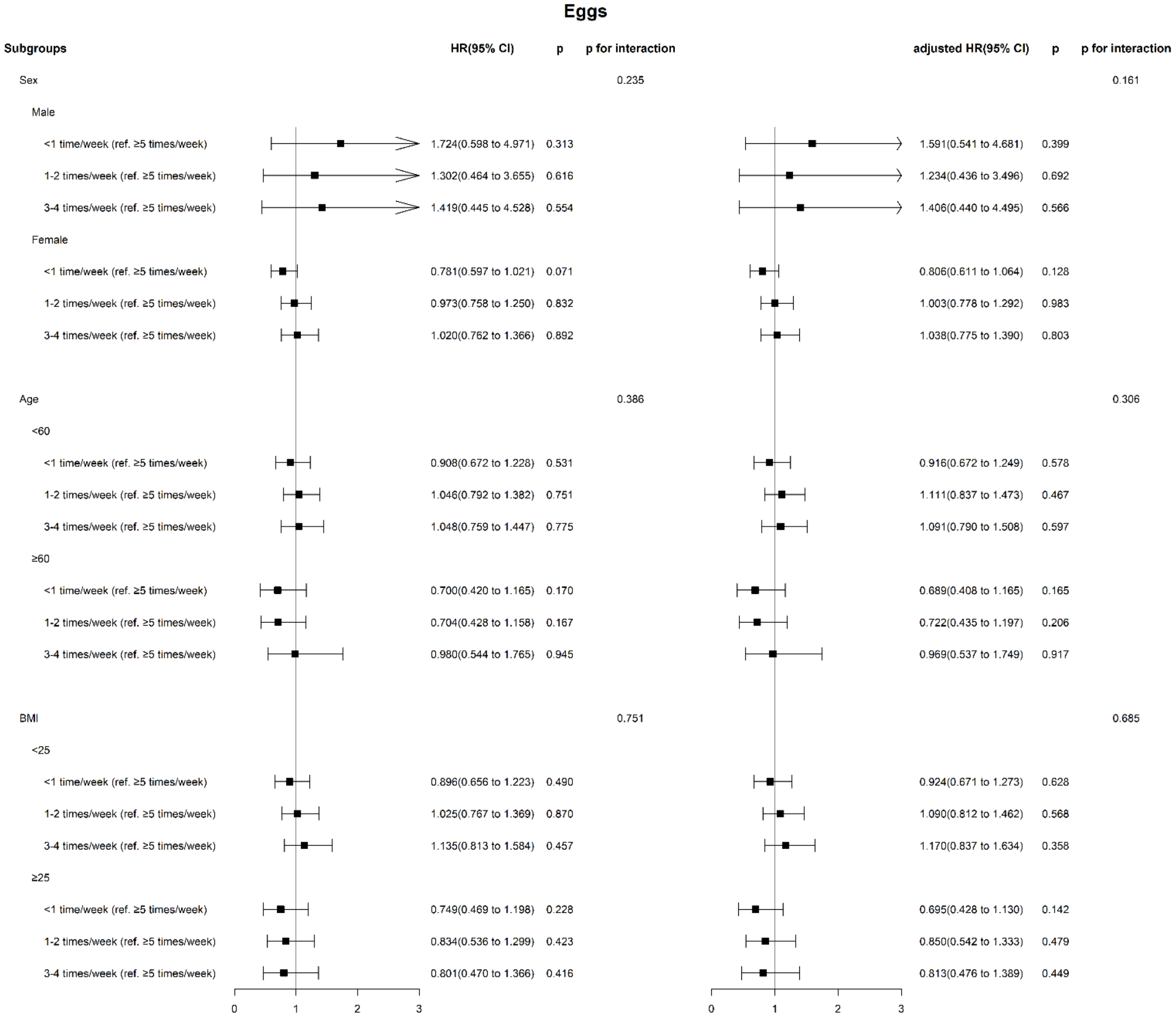

Supplementary Figure S2. Association between dairy products consumption and prevalence of thyroid cancer by sex, age, and obesity status.

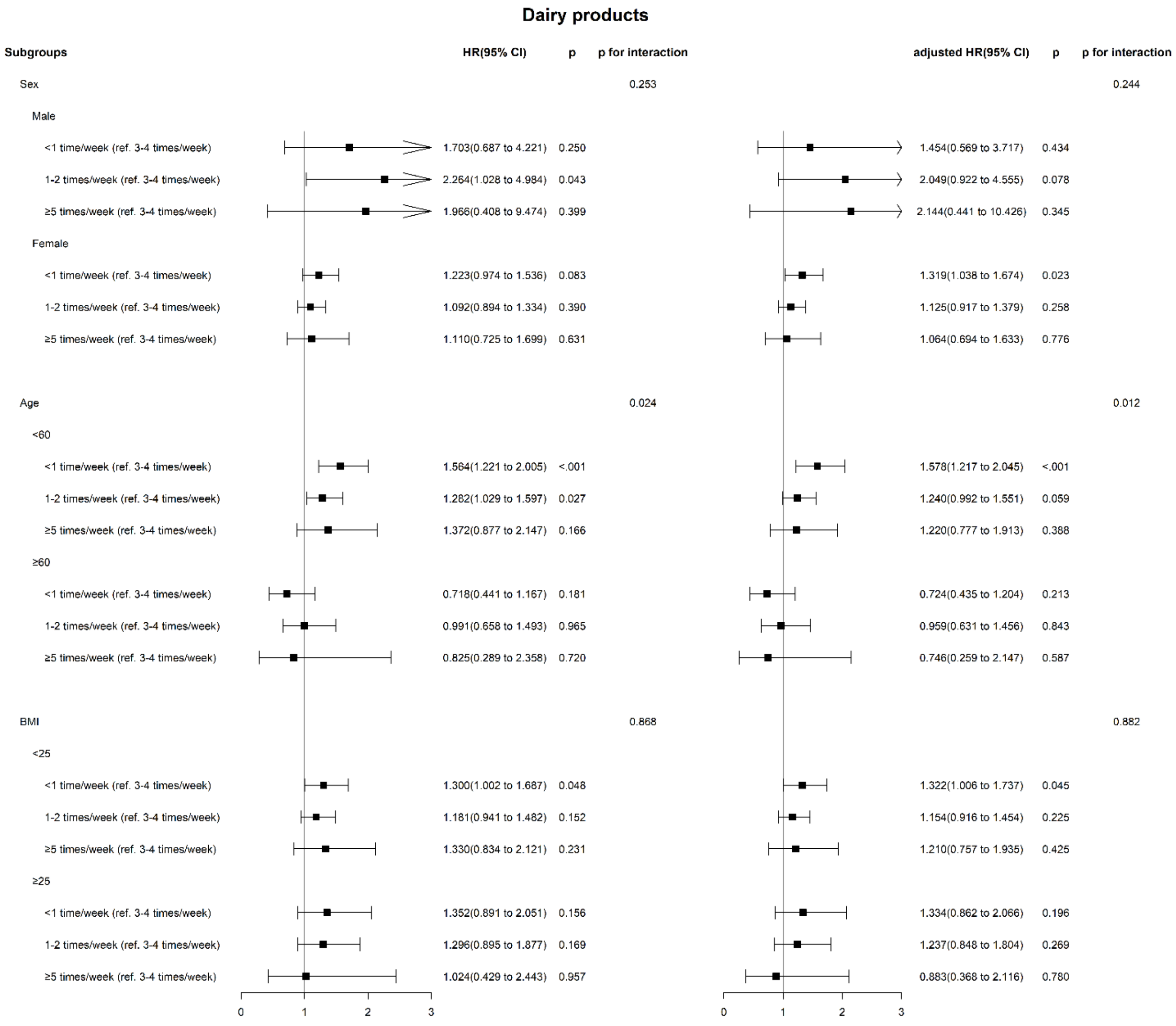

Supplement: Supplementary file 1 [file nutrients-16-01041-s001.zip › nutrients-2915530-supplementary.pdf]
